# The emerging role of dendritic cells in the tumor microenvironment: from antigen presentation to targeted immunotherapy

**DOI:** 10.1038/s41419-025-08180-0

**Published:** 2025-12-22

**Authors:** Zhiyuan Xie, Yingjun Fang, Xinhao Zhang, Yingshuai Fang, Ruiqi Li, Ying Guo, Yabing Yang, Shuaixi Yang, Lijie Song

**Affiliations:** 1 The First Clinical School of Medicine, Zhengzhou University, Zhengzhou, China; 2 College of Basic Medicine and Forensic Medicine, Henan University of Science and Technology, Luoyang, China; 3 Department of Colorectal Surgery, The First Affiliated Hospital of Zhengzhou University, Zhengzhou, China; 4 College of Life Science and Technology, North Henan Medical University, Xinxiang, China; 5 Department of Oncology, The First Affiliated Hospital of Zhengzhou University, Zhengzhou, China

**Keywords:** Antigen-presenting cells, Cell growth, Cancer microenvironment

## Abstract

Dendritic cells (DCs), as pivotal antigen-presenting cells (APCs), play crucial roles in initiating T cell-mediated antitumor immune responses, bridging innate and adaptive immunity while maintaining immune tolerance. With an in-depth understanding of DC biology and functions, numerous DC-targeted therapeutic approaches have been developed. An enhanced understanding of DC heterogeneity and DC cross-talk with other cells within the tumor microenvironment (TME), along with functional and metabolic remodeling mechanisms, may optimize DC-based cancer immunotherapies. This review focuses on the heterogeneity of the individual occurrence and function of DCs in tumors, elucidates the cross-talk between DCs and other cells in the TME, provides an in-depth understanding of the dysfunction and metabolic reprogramming of DCs in the TME, and summarizes existing DC-based anticancer therapies and novel therapeutic strategies, with the aim of providing new insight into the emerging role of DCs in future cancer immunotherapy.

## Facts

- DCs arise from distinct lineages with functional and state heterogeneity.
- DCs shape tumor progression via cross-talk in the tumor microenvironment.
- Tumor-driven DC reprogramming promotes immune tolerance.
- Targeting endogenous DCs offers promising avenues for cancer immunotherapy.
- DC-based vaccines and combination therapies show emerging clinical potential.

## Open Questions

- How can a unified classification system for DC subsets be established to clarify their developmental origins and interrelationships?
- Can specific cell–cell interactions involving DCs be therapeutically modulated to reverse immune suppression and restore antigen-presenting function in tumors?
- How can novel biomaterials, adjuvants, or delivery platforms be designed to selectively target DCs without impairing the physiological immune balance?

## Introduction

Dendritic cells (DCs) were first discovered by Ralph Steinman in 1973, a groundbreaking achievement that earned him the 2011 Nobel Prize [1, 2]. As professional antigen-presenting cells (APCs) bridging innate and adaptive immunity, DCs are significantly associated with better prognosis in cancer patients and enhanced clinical benefits from immune checkpoint blockade (ICB) [3]. DCs exhibit remarkable heterogeneity and are widely distributed in the skin, airways, intestines, lymphoid organs, and other tissues, where they can respond effectively to environmental stimuli [4].

The tumor microenvironment (TME), which comprises stromal and immune cells, critically influences tumor progression and therapeutic responses, immune cells within tumors include lymphocytes like T cells, B cells, and natural killer (NK) cells, as well as diverse myeloid cells such as granulocytes, monocytes, macrophages, and DCs [5]. TME composition varies across tumor types, and these cellular constituents are crucial in tumor initiation, progression, and metastasis [6]. Although DCs serve as central regulators in the TME by promoting antitumor T cell responses, the immunosuppressive TME can subvert DC effector functions through cell-cell contact and soluble mediators, ultimately driving DC phenotypic alterations, dysfunction, and tolerogenicity [7].

Here, we explore the origin and differentiation of DCs, with a focus on the heterogeneity of the individual occurrence and state of human DCs. We further elaborate on the cross-talk between DCs and other cells in the TME, as well as dysfunction and metabolic reprogramming in the TME. We also provide an emphasis on current DC-based cancer therapies and novel strategies, demonstrating the significant value of DCs as an emerging role in future anticancer immunotherapy.

## Initiating the journey: the origin and lineage evolution of DCs

**Fig. 1:**
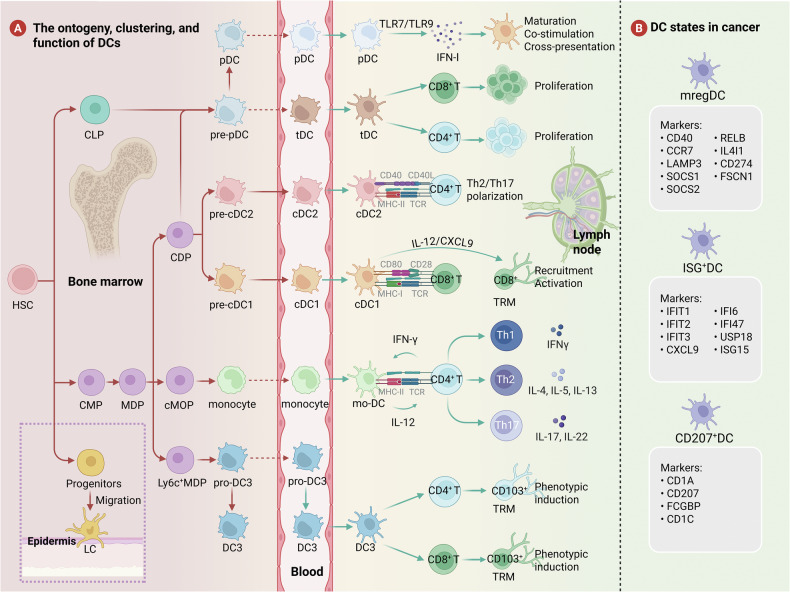
Functional and state heterogeneity of DC subsets. **A** Dendritic cells (DCs) originate from CD34⁺ hematopoietic stem cells (HSCs) and differentiate along distinct lineages into various subsets, including conventional DC1 (cDC1), cDC2, DC3, plasmacytoid DCs (pDCs), monocyte-derived DCs (mo-DCs), and transitional DCs (tDCs). Notably, pDCs can arise from both common myeloid progenitors (CMPs) and common lymphoid progenitors (CLPs), while tDCs, which exhibit features of both cDC2s and pDCs, develop from pre-pDCs. Langerhans cells (LCs), derived from precursor cells that migrate to the epidermis, significantly contribute to sustaining peripheral immune homeostasis. The distinct functional roles of these subsets highlight the extensive heterogeneity within the DC compartment. **B** In tumors, several mature DC states have been characterized, including mature DCs enriched in immunoregulatory molecules (mregDCs), interferon-stimulated genes (ISGs; ISG^+^ DCs), and CD207^+^ DCs. These states may arise from distinct developmental trajectories. Pre-pDC: pre-plasmacytoid dendritic cell; pre-cDC: pre-conventional dendritic cell; Pro-DC3: Lyz2^+^ DC progenitor; IFN: interferon; TRM: tissue-resident memory T cells; IL4I1: interleukin 4 induced 1; LAMP3: lysosomal associated membrane protein 3; SOCS: suppressor of cytokine signaling; IFIT: interferon-induced protein with tetratricopeptide repeats; USP18: ubiquitin-specific protease 18; FSCN1: fascin actin-bundling protein 1; FCGBP: IgGFc-binding protein. Created with BioRender.com.

As pivotal APCs, DCs are relatively low abundance in both the blood circulation and tissues [8] (Fig. 1A). Originating from multipotent CD34^+^ hematopoietic stem cells (HSCs) in the bone marrow, which act as precursors of multispectral progenitors that generate common myeloid progenitors (CMPs) and common lymphoid progenitors (CLPs), both retain the potential to differentiate into all DC subsets, of which only the subsets that express FLT3 can develop into DCs [9–11]. FLT3 is involved in cDC and pDC production, differentiation, and proliferation through interactions with FLT3L and is essential for the maintenance of DC dynamic homeostasis [12–15]. CMPs can differentiate into CX3CR1^+^ monocyte-dendritic cell progenitors (MDPs), which are common bone marrow precursors for macrophages and DCs and exhibit restricted lineage potential that excludes granulocytic, lymphoid, and NK cell differentiation [10, 14]. Subsequent developmental bifurcation generates downstream descendants, including common dendritic cell progenitor (CDP), common monocyte progenitor (cMOP), and Ly6C^+^ MDP populations [3, 12, 13].

CDPs are intermediate precursors for the production of conventional DCs (cDCs) and plasmacytoid DCs (pDCs), which can differentiate into different subsets of dendritic cells [13, 16]. During bone marrow differentiation, CDPs differentiate into pre-cDC1s, pre-cDC2s, and pre-pDCs [17]. Pre-cDC1s and pre-cDC2s migrate from the bone marrow to lymphoid and non-lymphoid tissues through the peripheral circulation, where they terminally differentiate into immature cDCs [9, 18, 19]. Notably, the expression of IRF8 is more critical for the development of cDC1s, whereas the development of cDC2s is more reliant on IRF4 expression [20]. Ly6C^+^ MDPs have lost monocyte potential and can give rise to pro-DC3s, which can differentiate to form DC3s either within the bone marrow or after migrating to peripheral tissues [12]. Pre-pDCs complete their maturation within the bone marrow before entering the circulation [15]. pDCs and cDCs share molecular mechanisms that allow FLT3L to control their differentiation, the formation of cDCs or pDCs from CDPs is associated with the specific E protein transcription factor TCF-4 (E2-2) and the DNA-binding protein inhibitor ID-2, and the inhibition of ID-2 expression favors the production of pDCs from CDPs, whereas the inhibition of TCF-4 expression favors the production of cDCs from CDPs [21, 22]. cMOPs can differentiate to form monocytes, and Ly6C^hi^ monocytes in the blood leave the bone marrow in a CCR2-dependent manner and can give rise to monocyte-derived DCs (mo-DCs) under inflammatory conditions as well as in tumors [12, 13, 19, 20]. The process of differentiation from Ly6C^+^ monocytes to mo-DCs is regulated by IRF4, PU.1 and MAFB [23].

In addition to having a shared origin with cDCs, pDCs have a unique differentiation pathway [24]. In another pathway, HSC-generated CLPs differentiate into pre-pDCs in the bone marrow, and pre-pDCs serve as precursor cells to generate pDCs [22]. Through the bloodstream, fully matured pDCs from the bone marrow disseminate to peripheral tissues [25]. pDCs exhibit several lymphocyte characteristics, but they share a developmental origin with cDCs, are specifically dependent on IRF8 transcription, can cross-present antigens upon activation, and influence T cell function, supporting the classification of pDCs as part of the DC lineage [18, 21, 26]. Furthermore, there are transitional DCs (tDCs) derived from the FLT3L-dependent pre-pDC pathway with a phenotype that spans the pDC-cDC2 continuum, which differentiate into mouse ESAM^+^ DC2s or human CD5^+^ DC2s in response to IRF4 [27].

Langerhans cells (LCs) are epidermally located macrophages that belong to the mononuclear lineage and have a different developmental pathway from FLT3-dependent conventional DCs, which can be derived from CD34^+^ HSCs, with fetal liver-derived non-inflammatory Ly6C^+^ monocytes serving as primary LC precursors and embryonic yolk sac myeloid progenitors are also precursors of LCs [9, 28]. The precursor cells pass through the bloodstream, migrate to the skin surface and differentiate into immature LCs [9]. Unlike many DC subsets, LCs are not replaced by bone marrow renewal metastases in their steady state but self-renew themselves in situ; however, at the same time, similar to monocytes and macrophages, their development is dependent on M-CSF [14]. Despite these macrophage-like properties, LCs are also categorized into a DC family because they share many of the characteristics of DCs, including morphology, common cell-surface markers, and significant T cell triggering potential [3, 14].

Under steady-state conditions, immature DCs in the blood and peripheral organs generate an immunosuppressive environment that inhibits self-reactive T cell activity and boosts the proliferation of regulatory T cells (Tregs), thereby maintaining peripheral immune tolerance to self-antigens [29–32]. Immature DCs have low major histocompatibility complex (MHC)-I and MHC-II, express low levels of costimulatory factors and chemokines, and do not secrete proinflammatory cytokines but exhibit potent antigen-capturing capacity [29]. DCs can undergo homeostatic maturation or immunogenic maturation induced by pattern recognition receptors (PRRs) [33]. Additionally, extensive cell-cell interactions and the production of soluble factors are involved in the induction and regulation of DC maturation [8]. Activated DCs experience alterations in gene expression and exhibit reduced capacity for antigen uptake and processing, while they express more MHC-II, CCR7, and costimulatory molecules, such as CD40, CD80, and CD86, and undergo CCR7-dependent migration to draining lymph nodes (dLNs) or the T cell zone in the splenic white pulp, where they ingest and process tumor antigens to T cells and other immune cells, resulting in the initiation of thus triggering effective cancer-specific immune responses [9, 31, 33, 34].

## Crossroads of differentiation: functional and state heterogeneity of DC subsets

DCs play pivotal roles in antitumor immune responses, with their subtype heterogeneity, transcriptional programs, tumor internal factors, and inflammatory environment collectively influencing their dual capacity to either promote or suppress tumor immunity [35]. DCs are classified into three major subsets on the basis of their functional and phenotypic markers: cDCs, pDCs, and mo-DCs [36]. cDCs are further subdivided into cDC1 and cDC2 subsets, which are defined by surface molecules and transcription factors [25]. In addition, LCs in the skin and mucosal epithelia contribute to peripheral immune homeostasis [14]. This phenotypic and functional plasticity enables DCs to dynamically regulate adaptive immunity according to microenvironmental demands, although it complicates precise classification [34] (Table 1). With the rapid advancement of single-cell RNA sequencing, the heterogeneity of DCs has been increasingly delineated. Emerging subsets include the DC_MKI67, defined by specific expression of MKI67; the transitional tDC, marked by AXL and CD5 expression; and three rare populations characterized by elevated levels of the transcription factors TRIM33, GTF2IRD1, and RUNX3 [37]. Moreover, conserved functional states that DCs acquire during maturation have been identified, extending beyond the boundaries of narrowly defined DC subsets [38]. Multiple DC functional states, including mature DCs enriched in immunoregulatory molecules (mregDCs), interferon-stimulated genes (ISGs; ISG^+^ DCs), and CD207^+^ DCs identified in various human and mouse tumors [8, 39] (Fig. 1).

**Table 1:** Overview of human DC subsets and functional states.

Subset/State	Transcriptional factor	Mouse markers	Human markers	Presence in vivo	Cancer type	Function
cDC1	FLT3L, BATF3, IRF8, ID2, NFIL3, ZBTB46	XCR1, CD24, CD8α, CD103, DEC205, CLEC9A	TLR3, CADM1, XCR1, CLEC9A, CD141	Thymus, spleen and lymph nodes	RCC, PCNSL, HCC, CTCL, MEL, BLCA	Cross presentation of antigens to activate CD8^+^ T cells
cDC2	FLT3L, RBPJ, IRF4, Notch2, KLF4, ZBTB46	CD11b, CD4, CD172a, CD301b	CD1c, CD11b, CD172a, CD1a, CD14, CD5	Thymus, spleen and lymph nodes	TGCT, MB, VS, GCTB, UVM, OS	Presenting exogenous antigens to activate CD4^+^ T cells
DC3	GM-CSF	CD45RB, CD11c, CD172a, CD16, CD32, CLEC12A	FLT3, HLA-DR, FCER1A, CD1c, CD163, CD14	Lymphoid tissues and blood	MEL, NSCLC, CRC	Induction of the CD103^+^ tissue-resident phenotype in CD4^+^ and CD8^+^ T cells
pDC	FLT3L, E2-2, IRF7, RUNX1	CD45R, CD317, SIGLECH, CD172a, CCR9, CXCR3, Ly6C, Ly6D	CD4, CD45RA, CD2, CD123, CD303, CD304, TLR7, TLR9	Bone marrow, lymphoid tissues, blood and tonsil	HNSC, CRC, CESC, ESCA, OV-FTC, MEL	IFN-Ⅰ production and low antigen presentation ability
tDC	FLT3L, TCF4	ESAM, CD135, CX3CR1	AXL, SIGLEC1, SLGLEC6, CD 22, HLA-DR, CD123, CD303, CD2, CX3CR1, CD 33, CD 5, CD 169	Blood	GEJ, MEL, ESCA, GIST, HNSC, BRCA	Ingestion of antigens, activation of antigen-specific naïve T cells, stimulation of T cell proliferation
mo-DC	CSF-1, GM-CSF, IL-4, IRF4, MAFB, PU.1	CD11b, CCR2, Ly6C, CD64, CD206, CD209, CD14	CD1c, CD11b, CD14, CD1a, CCR2, CD206, CD209	Skin, lung and intestine	NSCLC, CRC, MEL	Ingestion and cross presentation of antigens, limited migration ability and T cell stimulation ability
LC	PU.1, IRF4, BATF3, RUNX3, ID2	CD11c, MHC-II, CD207, CD11b, CD24, EpCAM	CD11c, HLA-DR, CD207, CD11b, CD24, CD1c, CD1a, EpCAM, CD83, CCR7	Skin	CSCC, MEL, HNSC	Maintain skin immune homeostasis
mregDC	IRF8, BATF3, FLT3L, ID2, NFIL3, RBPJ, IRF4, Notch2, KLF4	CCR7, FSC1, CCR7, CD274, PDCD1LG2	LAMP3, CCR7, CD274, FSCN1	Lymph nodes, skin, lung and intestine	CRC, ESCA, CSCC, STAD, BLCA, GEJ	Antigen presentation and immunoregulation
ISG^+^ DC	BATF3, FLT3L, ID2, NFIL3, RBPJ, IRF4, Notch2, KLF4	MHC-II, CD11c, CD11b, AXL	ISG15, IFI6, IFIT1	Blood, spleen and lymph nodes	MEL, Fibrosarcoma	Activation of CD8^+^ T cells and enhancement of their cytotoxicity
CD207^+^ DC	FLT3L, RBPJ, IRF4, Notch2, KLF4, ZBTB46	CD207, CD11c CD103	CD1c, CD207, CD1a	Skin	Meningioma, CSCC, HNSC	Associated with a favorable prognosis of tumors

*RCC* renal cell carcinoma, *PCNSL* primary central nervous system lymphoma, *HCC* hepatocellular carcinoma, *CTCL* cutaneous T-cell lymphoma, *MEL* melanoma, *BLCA* bladder cancer, *TGCT* Tenosynovial giant cell tumor, *MB* medulloblastoma, *VS* vestibular schwannoma, *GCTB* Giant cell tumor of bone, *UVM* uveal melanoma, *OS* osteosarcoma, *NSCLC* non-small cell lung cancer, *CRC* colorectal cancer, *HNSC* head and neck squamous cell carcinoma, *CESC* cervical squamous cell carcinoma, *ESCA* esophageal cancer, *OV-FTC* ovarian-fallopian tube cancer, *GEJ* gastro-esophageal junction cancer, *GIST* gastrointestinal stromal, *BRCA* breast cancer, *CSCC* cutaneous squamous cell carcinoma, *STAD* stomach adenocarcinoma, *GEJ* Gastro-esophageal junction cancer.

### DC subsets and functions

#### cDC1s

cDC1s are primarily used for antigen cross-presentation and CD8^+^ T cell activation and coordinate the CD8^+^ T cell response to ICB immunotherapy [40]. Lymphoid tissue-resident CD8α^+^ cDC1s and migratory CD103^+^ cDC1s have been identified in mouse models [41]. cDC1s are the only APCs capable of transporting intact antigens to lymph nodes and initiating tumor-specific CD8^+^ T cell responses, and they can also present exogenous antigens to CD4^+^ T cells through MHC-II molecules [42–44]. Beyond the activation of naïve CD8^+^ T cells, cDC1s serve as principal sources of IL-12 and CXCL9, orchestrating memory CD8^+^ T cells recruitment and reactivation within the TME [23].

#### cDC2s

cDC2s play crucial roles in exogenous antigen presentation, effectively presenting antigens associated with MHC-II to CD4^+^ T cells, thereby promoting Th2 and Th17 cell responses, and in some cases, Th1 and CD8^+^ T cell responses as well [40, 45]. cDC2s exhibit high heterogeneity, and their classification has been refined into different subgroups in recent years; these subgroups are typically divided into cDC2A and cDC2B based on T-bet expression [40]. Human cDC2As exhibit increased levels of amphiregulin, whereas human cDC2Bs exhibit a more proinflammatory phenotype [40]. In the most recent single-cell DC atlas, cDC2 cells have been classified into seven distinct transcriptional states: the cDC2_FCN1 subset, the cDC2_C1QC subset, the cDC2_CXCL9 subset, the cDC2_CXCL15 subset, the cDC2_CCR7 subset and cDC2_HSP subset, these subsets may possess distinct functional potentials in antitumor immune responses [37].

#### DC3s

Some studies categorize DC3s as a subgroup of cDC2s, suggesting that cDC2Bs and DC3s may represent overlapping populations [45]. However, related studies have revealed that DC3s originate from MDPs and develop along the IRF8^low^ SIRPA^+^ pathway with monocytes, whereas cDC2s are generated independently of monocytes, leading us to classify DC3s as a separate subset [46]. The DC3 subset may be crucial for tumor immunity and is capable of inducing autologous naïve CD4^+^ helper T cell responses and IL-17 production, as well as activating CD8^+^ T cells, with lower efficiency than cDC2s [35, 42, 47]. However, DC3s have the advantage of inducing the CD103^+^ tissue-resident phenotype in both CD4^+^ and CD8^+^ T cells [40]. Activated DC3s can upregulate CCR7 expression after toll-like receptor (TLR) stimulation and secrete T cell homing chemokines such as IL-12, CXCL9, and CXCL10 [48].

#### pDCs

pDCs are present in the bone marrow and all peripheral organs [13]. Compared with other DC subsets, pDCs exhibit lower CD11c and MHC-II expression levels with limited antigen-presenting capacity to T cells [23]. Specializing in antiviral immunity, pDCs induce IRF7-mediated IFN-I production and NF-κB-driven proinflammatory cytokine secretion through the recognition of TLR7 and TLR9 [49, 50]. IFN-I plays important roles in innate and adaptive antitumor immunity by enhancing cDC1 maturation, costimulatory molecule expression, and viral antigen cross-presentation [21, 34, 40]. Paradoxically, high tumor-infiltrating pDC density is correlated with poor prognosis in ovarian, cervical, head and neck cancers, melanoma, and breast cancer [51]. pDCs may exhibit immunosuppressive phenotypes and reduced production of IFN-α, enhancing their ability to induce Treg differentiation and leading to immunosuppressive responses within these TMEs [40, 42]. However, appropriately activated pDCs regain cross-presentation competence and transform into potent antitumor immune activators [52].

#### tDCs

scRNA-seq has identified a novel dendritic cell subset characterized by the expression of AXL, SIGLEC1, and SIGLEC6, designated tDCs or AS DCs [27, 53]. While pDCs share transcriptional similarities with pDCs and express pDC-associated markers (e.g., CD123, TLR7, and TLR9) and can respond to TLR7 and TLR9 agonists, tDCs lack pDC-defining functional genes [27, 53]. Unlike pDCs, tDCs are effective simulators of allogeneic CD 4^+^ and CD 8^+^ T cell proliferation (*P* < 0.01) and are slightly superior to cDC1s and cDC2s [53]. tDCs express functional markers of cDCs and can produce cDC1s and cDC2s in humans [27, 53]. However, tDCs have differentiated DC characteristics and can capture antigens and activate antigen-specific naïve T cells, so they are not pre-cDCs [27]. In summary, tDCs exhibit characteristics related to cDCs and pDCs but differ from cDCs and pDCs in that their gene expression spans cDC-like and pDC-like gene sets [53].

#### mo-DCs

mo-DCs, also referred to as inflammatory DCs (inf-DCs), originate from monocytes that are recruited to inflamed tissues during inflammation and are characterized by the highest levels of the pro-inflammatory cytokines IL-6 and IL-1β [15, 54]. mo-DCs can differentiate from peripheral blood CD14^+^ monocytes or CD34^+^ hematopoietic stem/progenitor cells under GM-CSF and IL-4 stimulation [25, 55]. The expression of DC surface markers such as CD1c, CD1a, and IRF4 in mo-DCs helps distinguish between mo-DCs and macrophage-like cells [35]. Mo-DCs exhibit relatively low immunogenicity. Although they display a pronounced capacity for tumor antigen uptake, promote the differentiation of CD4⁺ T cells into Th1, Th2, and Th17 phenotypes, and efficiently cross-present tumor antigens to CD8⁺ T cells, they express high levels of inducible nitric oxide synthase, leading to increased production of the T cell-suppressive molecule NO [20, 56]. Moreover, the lack of CCR7 expression restricts their migratory capacity, thereby limiting their ability to sustain effective immune responses in vivo [31, 42].

### Functional states of DCs in cancer

#### mregDCs

mregDCs have been identified in normal tissues, infections, autoimmune conditions, and various cancers, are also termed migratory DCs, CCR7^+^ DCs, or LAMP3^+^ DCs in some studies [8]. mregDCs can express maturation markers such as LAMP3, CD80, and CD83; the migration marker CCR7; the lymphocyte recirculation chemokines CCL19 and CCL21; low levels of TLR signaling genes and phagocytic receptors; and programmed cell death ligand 1(PD-L1) [57–59]. mregDC represent a mature state induced from cDC1s and cDC2s, in which mregDCs recruit T cells via the CCL19-CCR7 and CCL22-CCR4 axes, interact with CD4^+^ T cells, and secrete IL-12 to activate antitumor CD8^+^ T cells [8, 57, 60]. Among mregDCs, those derived from cDC1 exhibit a stronger capacity to promote antitumor immunity, whereas cDC2-derived mregDCs display weaker antigen-processing ability [37]. mregDCs are crucial for the survival and antitumor activity of cytotoxic T lymphocytes (CTLs) within the TME [61, 62]. mregDC is the cell state with the highest expression of CXCL16 (the ligand for CXCR6), CXCR6 is the most highly expressed chemokine receptor on tumor-infiltrating CTLs, and mregDCs can also express and transpresent the survival factor IL-15, which supports CTL survival [61]. On the other hand, mregDCs also express high levels of immune checkpoint–associated molecules and immunosuppressive genes, including PD-L1, PD-L2, and indoleamine 2,3-dioxygenase 1 (IDO1), which limit T cell activation and induce the generation of regulatory T cells (Tregs), thereby contributing to immunosuppression [47].

#### ISG^+^ DCs

ISG^+^ DCs can be identified under homeostatic and inflammatory conditions, and studies have suggested that ISG^+^ DC may represent a transitional state before DCs engage in the mregDC state [8]. Both cDC1s and cDC2s are involved in the formation of the ISG^+^ DC state [8]. The ISG^+^ DC state is characterized by a core set of ISGs, including CXCL9, CXCL10, IRF7, ISG15, and IFITM3, which are closely associated with interferon-induced signaling [8]. ISG^+^ DCs are found primarily within tumors, where tumor-derived IFN-β, a high-affinity member of the type I IFN family, induces ISG^+^ DCs to activate CD8^+^ T cells through “cross-dressing” of peptide-MHC (pMHC) complexes and their surface expression, enhancing cytotoxicity and promoting tumor regression [63].

#### CD207^+^ DCs

CD207^+^ DCs express CD1a and CD207, have been detected in a variety of cancer types, encompassing non-cutaneous malignancies [8]. cDC2s and LCs may contribute to the formation of the CD207^+^ DC state [3]. In colorectal and breast cancers, the substantial enrichment of CD207^+^ DCs correlates with better cancer prognosis [8].

### Spatial heterogeneity of DC subsets

The spatial heterogeneity of DC subsets is shaped by both tissue specificity and tumor context, and understanding these patterns is critical for optimizing immunotherapeutic targeting (Table 1). In humans, cDC1 and cDC2 predominantly localize to lymphoid organs such as the thymus, spleen, and lymph nodes, while mo-DCs are enriched in barrier tissues including the skin, lungs, and intestine [29]. In tumors, DC subsets show cancer type-specific abundance. cDC2 typically dominate across most malignancies, whereas cDC1 are comparatively enriched in non-small-cell lung cancer (NSCLC) [20]. MregDCs are markedly increased in esophageal, hepatic, gastric, and colorectal cancers but not in NSCLC [60]. Circulating DCs also differ between patients and healthy individuals: for instance, cDC2 are reduced in NSCLC and glioblastoma, increased in gastric cancer, while cDC1 decline in advanced melanoma [64–67].Furthermore, the relative proportions of DC subsets within tumors can change dynamically during tumor progression, and the total abundance of intratumoral DCs may also fluctuate, as demonstrated in the 3LL-R Lewis lung carcinoma model [20].

## Dialogue and game theory: the script of DC cross-talk in the TME

### TME and its immunosuppressive state

**Fig. 2:**
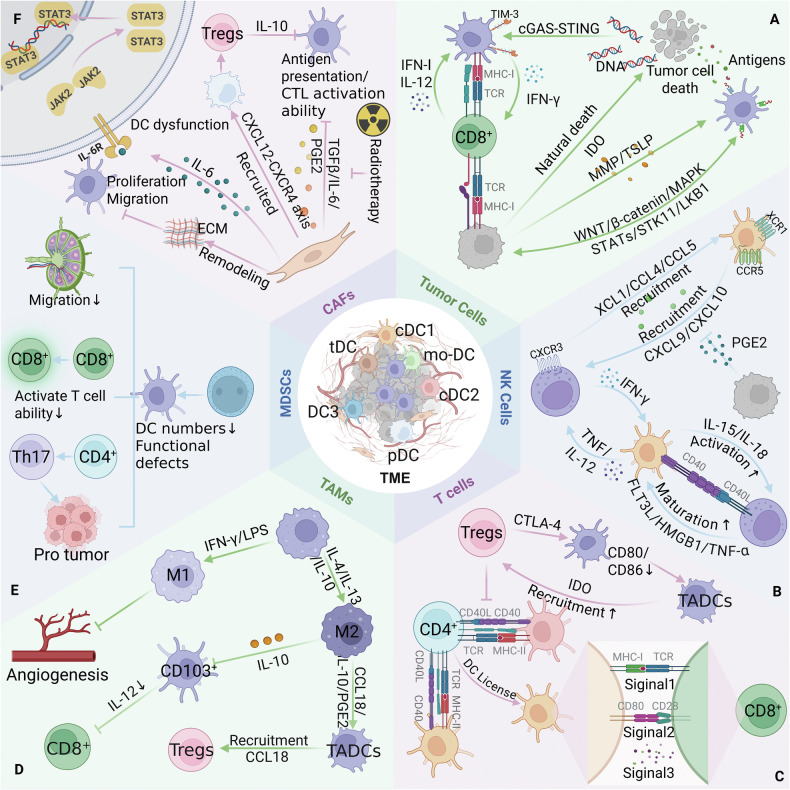
DCs engage in cross-talk with other cells in the TME. **A** Dying tumor cells release antigens and damage-associated molecular patterns (DAMPs), which subsequently activate dendritic cells (DCs). DCs recognize tumor-derived DNA via the cGAS-STING pathway, promoting the activation of CD8⁺ T cells. **B** Natural killer (NK) cells secrete XCL1, CCL4 and CCL5 to recruit cDC1s. In turn, cDC1s secrete CXCL9 and CXCL10 to recruit NK cells, while PGE2 secreted by tumor cells inhibits this process. **C** During T cell priming, DCs provide three key signals: the pMHC-TCR interaction (signal 1), costimulation (signal 2), and cytokine secretion (signal 3). cDC1s present antigens to CD4^+^ T cells, and through a mechanism termed DC licensing, acquire the ability to prime CD8^+^ T cells. **D** IL-10, CCL-18, and PGE2 derived from tumor-associated macrophages (TAMs) can affect DC maturation and function, promoting IL-18 production by tumor-associated DCs (TADCs), which subsequently recruit more regulatory T cells (Tregs). **E** Myeloid-derived suppressor cells (MDSCs) inhibit the migratory capacity of DCs and their ability to induce T cell activation. **F** TGF-β and PGE2 secreted by cancer-associated fibroblasts (CAFs) influence DC maturation and their capacity to activate cytotoxic T lymphocyte (CTL) responses. CAF-derived IL-6 impairs DC function through the IL-6-STAT3 signaling pathway. Additionally, CAFs affect the development and function of plasmacytoid DCs (pDCs) via the CXCL12-CXCR4 axis. TIM-3: T cell immunoglobulin and mucin domain 3; STAT: signal transducer and activator of transcription; STK11: serine/threonine kinase 11; MAPK: mitogen-activated protein kinase; LKB1: liver kinase B1; MMP: matrix metalloproteinase; TSLP: thymic stromal lymphopoietin; HMGB1: high mobility group box 1; TNF-α: tumor necrosis factor-alpha; PGE2: prostaglandin E2; LPS: lipopolysaccharide; ECM: extracellular matrix; JAK: janus kinase. Created with BioRender.com.

The TME constitutes a complex ecosystem of dynamically interacting cellular components, including neoplastic cells, immune populations, stromal cells, and the extracellular matrix (ECM) [40, 68]. The TME significantly influences the immune type, immune trajectory, and the fate of tumors [69]. The TME harbors diverse immunosuppressive cellular networks encompassing tumor-associated macrophages (TAMs), Tregs, myeloid-derived suppressor cells (MDSCs), and cancer-associated fibroblasts (CAFs), which collectively drive immune evasion and malignant progression through the establishment of an immunosuppressive microenvironment [70]. DC subsets at various maturation stages extensively infiltrate the TME [31]. As professional APCs that initiate and regulate immunity, DCs have their antitumor capacity profoundly impaired within the immunosuppressive TME [7]. A comprehensive understanding of their crosstalk with other cells in the TME provides critical insights for reversing immunosuppression, harnessing the therapeutic potential of DCs, and enhancing immunotherapy efficacy (Fig. 2).

### DCs cross-talk with tumor cells

DCs are professional APCs that can present tumor-associated antigens to T cells to trigger antitumor immune responses, while tumor cells secrete various immunosuppressive factors, including cytokines (e.g., IL-8, IL-10, and TGF-β), growth factors (e.g., VEGF), and hormones (e.g., PGE2), to impair DC activation, maturation, and functionality directly or modulate DC behavior via other immunosuppressive cells indirectly, ultimately driving immune evasion through DC tolerance induction and functional exhaustion [20, 51, 71]. Thymic stromal lymphopoietin and matrix metalloproteinase-2 produced by tumors can also affect DC function to induce a Th2 response [51]. In addition, active signaling pathways such as the WNT/β-catenin, MAPK, STAT, and STK11/LKB1 pathways critically regulate tumor cell-DC cross-talk within the TME [48, 51]. Tumor cell death modalities (spontaneous, therapy-induced, or immunogenic cell death [ICD]) release tumor antigens and damage-associated molecular patterns (DAMPs), triggering DC maturation, antigen processing and presentation, migration, and the activation of tumor antigen-specific T cells [8, 72]. Moreover, DNA released by tumor cells can stimulate the cGAS (cyclic GMP-AMP synthase)-STING (stimulator of interferon genes) pathway, enhancing type I IFN responses in DCs and activating CD8^+^ T cells [15] (Fig. 2A).

### DCs cross-talk with NK cells

NK cells can respond in the early stages of the antitumor immune response, whereas the T cell response is delayed because of the need for extensive clonal expansion from a limited number of antigen-reactive naïve precursors; both innate and adaptive immune responses are involved in antitumor immunity [73, 74]. Circulating human NK cells bifurcate into CD56^dim^CD16^hi^ and CD56^bright^CD16^lo^ subsets on the basis of surface CD56 (NCAM1) and CD16 (FCGR3A) expression [75]. CD56^dim^CD16^hi^ NK cells exert potent cytotoxicity via perforin and granzyme B secretion, whereas CD56^bright^CD16^lo^ NK cells produce substantial amounts of IFN-γ and other cytokines that are crucial for immunomodulation [75]. Although NK cells can kill circulating tumor cells, they have low cell killing efficiency in the TME [4]. NK-cDC1 cross-talk in the TME reciprocally enhances cDC1 recruitment, differentiation, maturation, and NK cell activation and cytotoxicity while promoting the development of Th1 cells and CTLs for the subsequent antitumor immune response [76]. Activated NK cells mediate tumor cell lysis and release of cellular debris, after which cDC1s process tumor antigens and migrate to LNs for cross-presentation to CD8^+^ T cells [44]. The cytotoxic function of NK cells and IFN-γ production are amplified by DCs through immunological synapse formation in a non-cell contact-dependent manner, such as through the secretion of cytokines IL-12 and IL-15 [77]. NK-derived IFN-γ serves as both a biomarker for durable antitumor responses and a predictor of long-term survival in cancer patients undergoing immunotherapy [78]. The differentiation and maturation of DCs are significantly influenced by NK cells. NK cells are key producers of the cDC1 differentiation factor FLT3L, which promotes DC differentiation, recruitment, and survival and controls DC levels in the TME [44, 79]. Moreover, NK cells can promote DC maturation through the secretion of cytokines such as IFN-γ, HMGB1, and TNF-α or by CD40-CD40L ligation, and in turn, mature DCs can release IL-12 and IL-18 to trigger NK cells to secrete cytokines that promote DC maturation [44, 77, 80]. DCs can release IL-15 and IL-18 to activate NK cells, which produce large amounts of TNF and IFN-γ, and TNF enhances costimulatory molecule expression on DCs synergistically with IFN-γ to promote the production of IL-12, which in turn can stimulate NK cells to secrete IFN-γ and boost the cytolytic activity of NK cells, forming a positive feedback loop [44, 76]. Moreover, the interaction between CD155 on cDC1s and CD226 on NK cells has been shown to contribute to NK cells activation [59].

cDC1s are essential for antitumor immunity, and their accumulation in mouse tumors often depends on NK cells [5]. NK cells can secrete chemokines such as XCL 1, CCL 4, and CCL 5, which recruit cDC1s to the TME via XCR1 and CCR5 [44]. Tumor-derived PGE2 can affect this process, preventing cDC1s from effectively migrating to the tumor site and thereby promoting immune evasion [5, 62]. cDC1s can also attract NK cells to the TME via CXCL9/CXCL10 (ligands for CXCR3) production and CXCR3-mediated chemotaxis [31]. Cross-talk between NKs and DCs in the TME contributes to the antitumor immune response; thus, targeting NK-DC crosstalk through targeted therapy and monoclonal antibody (mAb) therapy can increase cytotoxicity and improve the immunosuppressive microenvironment, thereby enhancing the antitumor immune response [44] (Fig. 2B).

### DCs cross-talk with T cells

T cells are categorized into CD4^+^ and CD8^+^ subsets on the basis of surface markers and functional specialization. CD4^+^ T cells are categorized into Th1, Th2, Th17, and follicular helper T (Tfh) cells and Tregs on the basis of their cytokine production, transcription factor expression, and expression of cell surface markers [29]. T cells coordinate pathogen-dependent immune responses through the production of different cytokines: Th1 cells mainly produce IFN-γ and express T-bet, controlling the proinflammatory phenotype; Th2 cells primarily generate IL-4, IL-5, and IL-13, with high expression of PLZF, coordinating the immunosuppressive phenotype; Th17 cells secrete IL-17 and IL-22, express RORγt; Tfh cells secrete IL-21 and IL-4, which are involved in B cell activation and differentiation; and Treg cells secrete IL-10, promoting immunosuppression [4, 81, 82]. In the cancer immune cycle, for T cells to generate effective anticancer immune responses, a series of events must be initiated and allowed to iterate through the cycle [83]. The antigen capture, processing, and presentation of DCs play important roles in activating effector T cells in this cycle [83]. During the activation of T cells, DCs provide three key signals: pMHC-T cell receptor (TCR) interaction (signal 1), costimulation (signal 2), and cytokine secretion (signal 3), the fate of T cells is determined by the strength, duration, and combination of these signals [63]. DCs load captured antigens onto MHC molecules to form pMHC complexes for surface display [35]. cDC1s activate CD8^+^ T cells through MHC-I cross-presentation, whereas cDC2s prime CD4^+^ T cells via MHC-II direct antigen presentation [35]. Upon CCR7/CCL21-dependent migration to tumor-draining LNs, DCs engage TCRs on T cells, triggering effector T cell activation [70]. Activated T cells can express CD40L and bind to the CD40 receptor on DCs [83]. Following CD40 ligation or TLR stimulation, cDC1s upregulate the expression of CD70 (a costimulatory ligand for CD27 expressed on T cells), promoting the differentiation of CD8^+^ T cell into effector and memory lymphocytes via the CD70-CD27 pathway [59]. CD8^+^ T cells eliminate tumor cells by recognizing tumor-associated antigens (TAAs) presented on MHC-I through TCRs overexpressed on their effector CTLs [51]. cDC1s can also present exogenous antigens to CD4^+^ T cells through MHC-II molecules, and following antigen recognition, antigen-specific CD4^+^ T cells can trigger DC activation, a process referred to as DC licensing, which facilitates the ability of cDC1s to effectively prime CD8^+^ T cells [59]. In this process, the interaction between CD40 on DCs and CD40L on CD4^+^ T cells is crucial [59]. DC-CD4^+^ T cell interactions drive Th subset differentiation: cDC1s preferentially induce Th1 cells, whereas cDC2s promote the polarization of Th2, Th17 and Tfh cells [34, 82]. DCs not only transport antigens to the LN but also facilitate local antigen-specific T cell activation and expansion within the TME [69, 84]. Tumor-infiltrating cDC1s recruit T cells via CXCL9/CXCL10 secretion (ligands for CXCR3) to guide T cell homing [48, 85]. After being triggered and activated by cDC1s, CD8^+^ T cells can secrete IFN-γ, which promotes the production of IL-12 by cDC1s in a non-canonical NF-κB-dependent manner; in turn, IL-12 can further stimulate cytotoxic CD8^+^ T cells, forming a positive feedback pathway [3, 70].

The nature of immune responses is determined by the balance between effector T cells and Tregs [83]. Tregs, defined as CD4^+^CD25^+^Foxp3^+^ T lymphocytes, prevent autoimmunity and modulate immune homeostasis under physiological conditions by suppressing IL-2 production, releasing adenosine, and secreting immunosuppressive cytokines, including IL-35, IL-10 and TGF-β [86]. Tregs constitute a major component of immune infiltration in the tumor stroma, and their presence correlates with adverse clinical outcomes of increased metastasis in many malignant tumors [51, 86, 87]. In the TME, Tregs can inhibit DC maturation by downregulating the expression of the costimulatory molecules CD80 and CD86 through CTLA-4 [51]. Immature DCs in the TME evolve into tumor-associated DCs (TADCs) characterized by elevated IL-10 and IDO expression, which promotes naïve CD4^+^ T cell differentiation into Tregs while reinforcing TADC immunosuppressive functions through IL-10 feedback loops to facilitate tumor progression [88, 89]. CXCR3 is a critical chemokine receptor for Treg accumulation and immunosuppression in tumors; its reduction promotes DC-CD8⁺ T cell interactions [90]. mregDCs recruit Tregs to the perilymphatic niche in the peripheral tumor stroma and promote their activation; the ensuing Treg-mregDC crosstalk restricts tumor-antigen trafficking to dLNs, thereby suppressing the initiation of adaptive antitumor immunity [91, 92] (Fig. 2C).

### DC cross-talk with TAMs

Macrophages, a heterogeneous myeloid population that can differentiate from monocytes under the induction of M-CSF, have diverse phenotypes and are influenced by the surrounding microenvironment to differentiate into two types: classically activated M1 (activated by IFN-γ/LPS) and alternatively activated M2 (activated by IL-4/IL-13/IL-10/glucocorticoids) [13, 93]. In the TME, M1 TAMs are generally characterized by their ability to inhibit angiogenesis and activate antitumor immunity, whereas M2 TAMs are characterized by their promotion of tumor growth, invasion, and metastasis [93, 94]. TAMs that inhibit angiogenesis and activate antitumor immunity are typically defined as M1 TAMs, Moreover, there is evidence that TAMs do not follow simple M1-M2 polarization in vivo [6, 95, 96]. TAMs have immunosuppressive activity, and the colocalization of immunosuppressive TAMs with DCs inhibits antigen presentation and infiltration of DCs, while TAMs can also produce high levels of IL-10 and low levels of IL-12, leading to DC dysfunction [93, 94, 97]. In breast cancer, TAM-secreted IL-10 suppresses CD103^+^ DC IL-12 production, impairing CD8^+^ T cell responses during cyclophosphamide chemotherapy [97]. In the TME, CCL18 is produced mainly by TAMs, which can directly recruit immature DCs into the TME, where these immature DCs are induced to form immunosuppressive TADCs under the influence of CCL18, IL-10, and PGE2 secreted by TAMs [88]. Immature DCs can also secrete CCL18, recruiting more immature DCs and Tregs, and the immunosuppressive TME is maintained by the combined action of these immunosuppressive cell populations [88] (Fig. 2D).

### DC cross-talk with MDSCs

MDSCs are immature heterogeneous myeloid populations with immunoregulatory functions and are usually categorized into monocytic MDSCs (M-MDSCs) and polymorphonuclear MDSCs (PMN-MDSCs) [4]. In cancer patients, MDSCs are overproduced in the bone marrow and aberrantly accumulate in the tumor stroma, with hypoxic TME conditions further promoting their recruitment [87]. In contrast to MDSCs in peripheral lymphoid organs, TME-resident MDSCs exhibit an enhanced inhibitory phenotype and upregulated expression of inhibitory molecules, and they can promote tumor growth through various mechanisms [4, 93]. Within tumors, immunosuppressive MDSCs can differentiate into TAMs or inhibitory DC subsets [98]. MDSC-DC cross-talk influences the immune activity of DCs, potentially explaining the reduced number of mature DCs and functional deficits observed in cancer patients [93, 98]. Melanoma studies revealed that MDSCs can reduce DC antigen uptake, block DC migration, and promote DC production of the proinflammatory cytokine IL-23, which induces the formation of Th17 cells downstream, thereby suppressing immune surveillance and promoting metastasis by affecting both innate and adaptive immunity [93]. MDSCs can impair DC function by secreting IL-10, which suppresses DC production of IL-12 and reduces DC-mediated T cell activation, thereby promoting tumor progression [98]. Additionally, MDSCs can also exhibit tumor-reducing properties according to some experimental evidence, which is likely related to their population and effects on NK cells [93] (Fig. 2E).

### DC cross-talk with CAFs

CAFs in most human solid tumors constitute three predominant subsets: myofibroblastic CAFs, inflammatory CAFs, and antigen-presenting CAFs [69, 99]. CAFs critically regulate ECM maintenance, desmoplasia, angiogenesis, invasion, immunosuppression, and chemoresistance [86]. Within the TME, CAF-derived soluble mediators (e.g., TGFβ, IL-6, and PGE2) can impair DC maturation and suppress DC antigen-presentation capacity and the ability to activate CTL responses, whereas radiotherapy can reverse this inhibitory effect [100]. CAFs abundantly produce CXCL12, which governs the migration and recruitment of DCs within tumors via the CXCL12-CXCR4 axis [100]. CXCL12 attracts mature pDCs that stimulate the development of Tregs to produce IL-10, thereby inhibiting DCs to activate tumor-specific T cell responses and promote tumor progression [100]. Under high-CXCL12 conditions, pDCs synergize with CAF-derived VEGF through TNF-α and IL-8 secretion to reduce tumor angiogenesis [100]. CAF-derived IL-6 can reduce the expression of MHC-II on the surface of DCs via the IL-6-STAT3 signaling pathway, thereby suppressing CD4^+^ T cell-mediated immune responses [100]. CAFs secrete WNT2, which inhibits DC-mediated antitumor T cell responses through the SOCS3/p-JAK2/p-STAT3 signaling pathway [101, 102]. Additionally, CAFs remodel the ECM into dense fibrotic stroma, which impedes DC proliferation and migration, blocks T cell infiltration, and recruits MDSCs [72] (Fig. 2F).

## Silent sentinels: metabolic reprogramming of DCs in the TME

As pivotal APCs, effective antitumor T cell responses depend on the differentiation, infiltration, migration, and functional status of DCs in the TME [71]. However, the TME is often characterized by hypoxia, accumulation of extracellular adenosine, lactate buildup, and reduced pH, which collectively impair DC activation, maturation, and function, leading to a tolerogenic phenotype [71, 98, 103]. Tolerogenic DCs are characterized by impaired maturation, downregulation of costimulatory molecules and proinflammatory cytokines, upregulation of inhibitory molecules and anti-inflammatory cytokines, and increased fatty acid oxidation (FAO) and can induce Treg generation and inhibit T cell proliferation and activation [104, 105]. DCs respond to intrinsic and extrinsic signals by establishing a complex network of metabolic pathways, including glycolysis, oxidative phosphorylation (OXPHOS), and fatty acid metabolism [106]. Within the TME, tumors compete for critical nutrients (e.g., glucose and amino acids), reprogramming core metabolic pathways in immune cells and impairing antitumor immunity [107, 108]. Metabolic reprogramming of DCs has become an important mechanism of immune tolerance, driving the occurrence of tumor-mediated immune evasion [103].

### Carbohydrate metabolism reprogramming of DCs in the TME

In highly proliferative cells or tumor cells, the metabolic spectrum shifts from OXPHOS to aerobic glycolysis, known as the Warburg effect [109]. Although mature DCs cannot proliferate upon activation, they rely on glycolysis and the pentose phosphate pathway to maintain their energy and membrane integrity, whereas immature and tolerogenic DCs predominantly utilize OXPHOS and FAO as their metabolic pathways [105, 109, 110]. The shift in carbohydrate metabolism ensures that DCs have sufficient energy to sustain various biosynthetic and immune functions [111]. In the TME, glucose deprivation of TADCs by growing tumors activates AMPK in TADCs, which, together with lactate accumulation, may lead to the inhibition of glycolysis and the upregulation of OXPHOS in TADCs [112–114]. Glycolysis inhibition impairs DC shape maintenance, CCR7 oligomerization, DC migration to dLNs, and the production of costimulatory molecules and proinflammatory cytokines, ultimately affecting DC antigen presentation and T cell stimulation [115, 116]. The competitive glucose uptake by activated T cells further exacerbates glucose insufficiency in DCs [36]. Subsequently, DCs gradually adapt to substantial glucose consumption in the TME by shifting their metabolic profile toward OXPHOS, which can cause DCs to exhibit tolerance characteristics [117]. Notably, DC subsets exhibit distinct metabolic features in response to TLR stimulation: cDCs primarily utilize glycolysis, whereas pDCs mainly utilize OXPHOS, reflecting the unique biological functions of different DCs; here, we focus mainly on cDCs as an example [117, 118]. Therefore, reducing the dependence of DCs on glycolysis or selecting subsets less susceptible to glycolytic inhibition represents a potential strategy to enhance antitumor immunity.

### Lipid metabolism reprogramming of DCs in the TME

Under steady-state conditions, appropriate levels of fatty acid synthesis (FAS) and fatty acids (FAs) are critical for DC maturation, proinflammatory cytokine expression, and the acquisition of immunogenicity [111]. Lipid homeostasis constitutes a critical metabolic checkpoint of DC function, frequently exploited by tumors to suppress antitumor immunity [8]. In breast cancer, upregulation of fatty acid synthase results in high extracellular FA accumulation, which promotes FA deposition within DCs [119]. This, in turn, diminishes their antigen-processing ability, downregulates the costimulatory molecule CD86, and induces excessive expression of the tolerogenic cytokine IL-10, ultimately reducing their ability to activate T cells and promoting tumor immune tolerance—a phenomenon predominantly observed in cDCs but not in pDCs [36]. In addition, uptake of oxidized lipids induces ER stress via the IRE1/XBP1 axis, driving excessive lipid synthesis and diminishing DC immunogenicity [48]. Enhanced FAO further amplifies IDO1 activity and Treg generation while shifting DC metabolism from glycolysis to oxidative phosphorylation to reinforce FAS, maintaining a balance between FAS and FAO, these adaptations may further contribute to tumor immune tolerance [36, 114, 120]. In summary, therapeutic strategies aimed at inhibiting fatty acid synthase in tumor cells or abrogating XBP1 signaling in DCs may restore DC immunogenicity and represent promising avenues for cancer immunotherapy.

### Amino acid metabolism competition of DCs in the TME

In the TME, amino acids such as tryptophan, arginine, and glutamine are biologically essential for both tumor cells and immune cells. The expression of Arginase 1 and IDO in TADCs leads to arginine and tryptophan depletion in the TME and suppresses the CD8^+^ T cell response and survival [114]. IDO, the rate-limiting enzyme in tryptophan catabolism, converts L-tryptophan to L-kynurenine in DCs, impairing CTL activity and enhancing Treg and TAM functions while simultaneously hindering DC maturation [70]. Tregs can induce other DCs to express IDO1 through interaction with CTLA-4, creating a metabolic-immunosuppressive positive feedback loop that exacerbates immune suppression and promotes cancer immune evasion [36]. Glutamine is a dominant amino acid that promotes the function of cDC1s and can trigger their antitumor immunity [121]. In the TME, tumor cells outcompete DCs for glutamine uptake, with tumor-mediated glutamine deprivation impairing DC activation and subsequent CD8^+^ T cell immune responses, thereby facilitating immune escape [121]. Meanwhile, glutamine catabolism by tumors fosters an acidic TME, thereby limiting DC antigen uptake and compromising the stability of antigen-MHC-I complexes [36]. Therapeutic targeting of glutamine metabolism represents a promising strategy to increase cancer treatment efficacy [121].

### The acquisition of vitamin A metabolic capacity by DCs in the TME

Retinoic acid (RA) is an active metabolite of vitamin A and plays an important role in regulating the immune response [122]. Studies have revealed that tumors in the TME activate the β-catenin/TCF pathway in DCs, enabling them to metabolize vitamin A into RA [122]. RA directs the differentiation of monocytes in tumors into immunosuppressive macrophages rather than immunostimulatory DCs by suppressing the DC-promoting transcription factor IRF4 [19, 123]. RA can also suppress the key glycolytic enzyme pyruvate kinase M2 by inducing SOCS3 expression in DCs, thereby leading to impaired antigen-presenting function [122, 124].

## Rebooting the defense line: cancer immunotherapy strategies targeting DCs

**Fig. 3:**
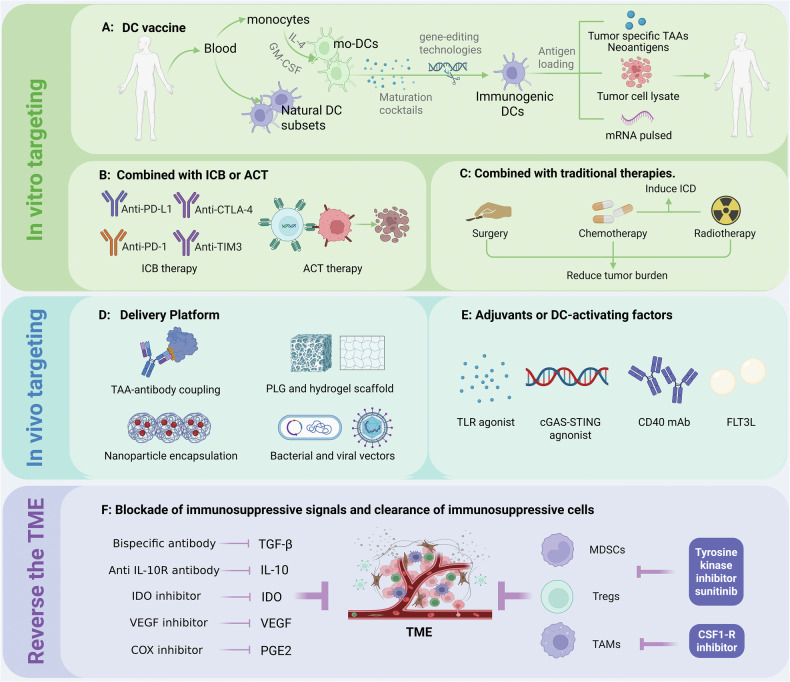
DC-based cancer therapy strategies. Current dendritic cell (DC)-based cancer immunotherapeutic strategies include DC vaccines, either alone or in combination with immune checkpoint blockade (ICB), adoptive cell transfer (ACT), and conventional therapies. In parallel, platforms and adjuvants targeting endogenous DCs have shown significant potential in enhancing antitumor immunity. Furthermore, strategies aimed at reversing the immunosuppressive tumor microenvironment can improve the efficacy of DC-based immunotherapies. PLG: poly-lactide-co-glycolide. Created with BioRender.com.

DCs are valuable targets for immunotherapy, with DC-based strategies demonstrating significant potential across diverse malignancies [29]. Current strategies targeting DCs, including DC vaccines and their combination therapies, genetic engineering, and targeting of endogenous DCs, not only provide new insights for cancer immunotherapy but also establish a technical foundation for individualized precision medicine. Tumor-induced immune suppression and resistance significantly impact the efficacy of therapeutic cancer vaccines [72]. Therefore, this section will also cover strategies to reverse the immunosuppressive microenvironment to increase the efficacy of DC-based immunotherapies (Fig. 3).

### DC vaccines

DC vaccines represent a vaccination approach involving ex vivo infusion of mature DCs loaded with tumor antigens into patients [52]. Despite their demonstrated immunogenicity and safety in numerous clinical studies and trials, their therapeutic efficacy remains limited, with objective response rates rarely exceeding 15%, restricting their value as clinically effective cancer immunotherapies [3]. Most current preclinical and clinical DC vaccines use mo-DCs generated ex vivo from monocytes under IL-4 and GM-CSF stimulation rather than being isolated from peripheral blood [3]. However, compared with ex vivo-generated DCs, natural DC subsets possess more powerful antigen-presenting abilities and thus can serve as foundational platforms for next-generation DC vaccine development [125]. Because of the extremely low numbers of cDC1s in the peripheral circulation, initial clinical studies of natural DC vaccines have utilized the more abundant cDC2 or pDC subsets [55, 125, 126]. After being obtained ex vivo, DCs are typically manipulated via maturation cocktails (TNF-α, IL-1β, polyI:C, IFN-α, and IFN-γ) or gene-editing technologies (RNA interference, viral transduction, and CRISPR/CRISPR-associated protein9) to increase their immunogenicity and promote T cell responses [52, 55, 127].

The suboptimal selection of target antigens is one of the reasons for the limited success of DC vaccines, and advancements in neoantigen prediction algorithms and large-scale parallel genome sequencing have enhanced this process [63]. TAAs are most commonly used to load patient-derived DCs for the preparation of tumor vaccines [128]. Sipuleucel-T, the first FDA-approved autologous DC vaccine loaded with prostate acid phosphatase antigen, improved survival in castration-resistant prostate cancer patients (HR = 0.78; *P* = 0.03) in a phase III trial (NCT00065442) [129]. However, antigens loaded onto DC vaccines often induce immune tolerance in refractory patients [130]. In recent years, personalized DC vaccines based on neoantigens have emerged as a novel direction in cancer immunotherapy by suppressing tumor escape [72, 130]. Neoantigens are antigens generated in tumors through mechanisms such as nonsynonymous somatic mutations in coding regions and can spontaneously and postimmunotherapy elicit robust T cell responses against these antigens due to the absence of central tolerance in the host [72]. A recent study demonstrated that combined administration of neoantigen and in situ cancer vaccines elicits tumor-specific immunity and shows clinical promise [131].

The loading of a single antigen can lead to tumor escape variants due to selective pressure and high mutation rates, thereby promoting tumor evasion [128]. To address these limitations, therapeutic strategies utilizing whole tumor cells or tumor cell lysates as antigen sources for DC loading have demonstrated clinical benefits in melanoma, non-Hodgkin lymphoma, and renal cancer, particularly in indications where surgery can be part of the treatment [128]. Glioblastoma is the most common and aggressive primary malignant brain tumor [132]. A prospective multicenter placebo-controlled randomized phase III trial reported that DCVax-L (an autologous tumor lysate-loaded DC vaccine) improved median and long-term survival in both newly diagnosed and recurrent glioblastoma patients, but the trial’s unique design and methodological constraints limit the reliability of the results, necessitating further prospective validation studies [133, 134].

Breakthroughs in mRNA technology have provided DC vaccines with more flexible antigen loading modalities. mRNA is introduced into DCs via lipid nanoparticles or electroporation, enabling the natural expression, processing, and presentation of encoded proteins, the safety and feasibility of which have been demonstrated in phase I studies of metastatic renal cell carcinoma, melanoma, and glioblastoma [128, 135, 136]. Recently, a study using Wilms tumor protein (WT1)-mRNA-electroporated autologous DC (WT1-mRNA/DC) as an adjuvant vaccine demonstrated safety and feasibility in patients with various solid tumors, with further clinical studies ongoing [137]. Antigenic mRNA can be electroporated combined with the TriMix formulation, which includes mRNA-encoded adjuvants such as constitutively active TLR4, CD40L and CD70 [138]. DCs matured by TriMix electroporation can convert Tregs into Th1-like cells while increasing IL-12 secretion, thereby reducing Treg-mediated suppression of CD8^+^ T cells both in vivo and in vitro [138]. Currently, DC-tumor cell fusion vaccines and tumor small extracellular vesicle-based DC vaccines have achieved significant breakthroughs in cancer immunotherapy, and the lentiviral construct SmartDC, which expresses IL-4, TRP2 (a melanoma antigen), and GM-CSF, has also demonstrated promise in this field [15, 130, 138].

### DC vaccines in combination with ICB and adoptive cell transfer (ACT) therapy

ICB can reprogram the interaction between the STAT3 and STAT5 transcriptional pathways in DCs, activate T cell immunity, and disrupt cancer cell–mediated impairment of immune surveillance [139]. However, the clinical efficacy of ICB monotherapy remains limited, contingent upon checkpoint molecule expression, tumor mutational burden, and host immune status [140, 141]. DCs are crucial in anticancer immunity, and their ability to prime, restimulate, and sustain tumor-specific T cells underpins the efficacy of ICB [63]. Combinatorial strategies integrating DC vaccines with checkpoint inhibitors enhance antitumor immunity by strengthening DC-T cell interactions, demonstrating effective antitumor activity in various cancer models [142, 143]. Numerous clinical trials are currently recruiting or being completed (Table 2). Compared with ipilimumab monotherapy, the mRNA-based DC vaccine TriMix-DC, in combination with ipilimumab (TriMixDC-PDMMEL IPI), elicits more effective CD8^+^ T cell responses in stage III or IV melanoma patients [144]. TIM-3 blockade can enhance the DC-mediated coordination of innate and adaptive immune responses [145]. Preclinical data support TIM-3 blockade as a valuable combinatorial target [70]. ACT has achieved clinical success in patients with B cell malignancies [146]. Successful adoptive T cell therapy relies on the presence of cDC1s within the tumor to provide T cell homing chemokines and support adoptively transferred T cell expansion via CD40- and CD70-dependent mechanisms [48]. ACT enhances the efficacy of DC-based immunotherapy, whereas DCs can improve the efficiency of ACT [15]. A Phase I trial (NCT01946373) demonstrated improved clinical responses in stage IV melanoma patients receiving ACT of tumor-infiltrating lymphocytes combined with DC vaccination, compared to ACT monotherapy [147].

**Table 2:** Ongoing and completed clinical trials involving the combination of DC vaccines and ICBs.

NCT Number	Conditions	Phase	Interventions	Status
NCT02529072	Recurrent Grade III and Grade IV Brain Tumors	Phase Ⅰ	DC vaccine + Nivolumab	Completed
NCT03092453	Melanoma	Phase Ⅰ	DC vaccine + Pembrolizumab	Completed
NCT03035331	NHL	Phase Ⅰ/Ⅱ	DC therapy + cryosurgery + Pembrolizumab	Completed
NCT03879512	Childhood Glioblastoma	Phase Ⅰ/Ⅱ	DC vaccine + metronomic cyclophosphamide + Nivolumab + Ipilimumab	Completed
NCT03325101	Metastatic or Unresectable Melanoma	Phase Ⅰ/Ⅱ	DC therapy + cryosurgery + Pembrolizumab	Completed
NCT01302496	Stage III or IV Malignant Melanoma	Phase Ⅱ	TriMix-DC + Ipilimumab	Completed
NCT01804465	Prostate Cancer	Phase Ⅱ	Sipuleucel-T + Ipilimumab	Completed
NCT02677155	Follicular Lymphoma	Phase Ⅱ	DC vaccine + Pembrolizumab + Rituximab + Radiotherapy	Completed
NCT06329908	Advanced Lung Cancer Resistant to ICIs	Early Phase Ⅰ	DC vaccine + ICIs	Recruiting
NCT05631886	Local Advanced/Metastatic Solid Tumors or R/R Lymphomas	Phase Ⅰ	TP53-EphA-2-CAR-DC + anti-PD-1 antibody/anti-CTLA4 antibody	Recruiting
NCT05631899	Local Advanced/Metastatic Solid Tumors	Phase Ⅰ	KRAS-EphA-2-CAR-DC + anti-PD-1 antibody/anti-CTLA4 antibody	Recruiting
NCT04201873	Recurrent Glioblastoma	Phase Ⅰ	ATL-DC vaccine + Pembrolizumab	Recruiting
NCT02070406	Unspecified Adult Solid Tumor, Protocol Specific	Phase Ⅰ	DC vaccine + gene-modified T-cells + Ipilimumab	Terminated
NCT03546426	PD-L1 Negative Advanced Mesothelioma	Phase Ⅰ	Autologous DC vaccine + Pembrolizumab	Active, not recruiting
NCT05457959	Diffuse Hemispheric Glioma, H3 G34-Mutant	Phase Ⅰ	Peptide-pulsed DC vaccine + Nivolumab + Ipilimumab	Withdrawn
NCT05765084	Malignant Pleural Mesothelioma	Phase Ⅰ/Ⅱ	WT1/DC vaccine + Atezolizumab	Recruiting
NCT04487756	Extensive-stage SCLC	Phase Ⅰ/Ⅱ	Autologous DC vaccine + Atezolizumab	Active, not recruiting
NCT04888611	Recurrent Glioblastoma	Phase Ⅱ	GSC-DCV + Camrelizumab	Unknown
NCT03782064	Multiple Myeloma	Phase Ⅱ	DC/MM Fusion vaccine (DC/MM vaccine) + Nivolumab	Terminated
NCT06751849	Advanced NSCLC	Phase Ⅱ	DC vaccine + PD-1 Inhibitor + Radiotherapy	Recruiting
NCT06522919	mCRC	Phase Ⅱ	DC vaccine + Pembrolizumab + FTD/TPI + Bevacizumab	Recruiting
NCT04912765	HCC and CRLM	Phase Ⅱ	Neoantigen DC vaccine + Nivolumab	Recruiting
NCT03406715	Relapsed SCLC	Phase Ⅱ	Ad.p53-DC + Nivolumab + Ipilimumab	Terminated
NCT04348747	TNBC or HER2^+^ BC or HR^+^ BC Brain Metastasis	Phase Ⅱ	Anti-HER2/HER3 DC vaccine + Pembrolizumab	Recruiting

Immune checkpoint inhibitors: PD-1 inhibitor: Nivolumab, Pembrolizumab, Camrelizumab. PD-L1 inhibitor: Atezolizumab. CTLA-4 inhibitor: Ipilimumab.

*NSCLC* non-small cell lung cancer, *HCC* hepatocellular carcinoma, *CRLM* colorectal liver metastasis, *SCLC* small cell lung cancer, *NHL* non-Hodgkin lymphoma, *mCRC* metastatic colorectal cancer, *TNBC* triple negative breast cancer, *HER2*^+^ *BC* HER2^+^ Breast Cancer, *HR*^+^ *BC* Hormone Receptor Positive Breast Cancer, *ICIs* Immune Checkpoint Inhibitors, *TP53-EphA-2-CAR-DC* Autologous EphA2-targeting CAR-DC vaccine loaded with TP53 mutant peptide, *KRAS-EphA-2-CAR-DC* Autologous EphA2-targeting CAR-DC vaccine loaded with KRAS mutant peptide, *ATL-DC vaccine* Autologous tumor lysate pulsed DC vaccine, *GSC-DCV* Glioblastoma Stem-like Cell Antigens-primed DC Vaccines, *FTD/TPI* Trifluridine/Tipiracil, *Ad.p53-DC* DC Based p53 Vaccine.

### DC vaccines in combination with other cancer therapies

Conventional therapies, including chemotherapy, radiotherapy, and surgery, reduce the tumor burden, and the use of DC vaccines as adjuvants and/or consolidation therapies in the early stages of the disease or in patients who can receive the aforementioned therapies has shown beneficial outcomes in cancer treatment and postoperative recurrence prevention [3]. Radiotherapy and chemotherapy can convert immunologically “cold” tumors to “hot” tumors through ICD induction, enhancing tumor antigen immunogenicity and subsequent vaccination efficacy [128].

### Therapies targeting endogenous DCs

#### Carriers for targeting endogenous DCs

Ex vivo manipulation of DC vaccines faces significant limitations, but selectively targeting antigens and/or immunostimulatory molecules to specific DCs in vivo through various strategies and directly activating natural DC subsets at multiple sites within the body have emerged as promising therapeutic approaches [34, 148]. Current approaches include antigen conjugation with DC-targeting antibodies, antigen/adjuvant encapsulation in biodegradable materials or nanoparticles, and microbial vector utilization [149]. The conjugation of TAAs to DC-specific antibodies enhances cross-presentation and elicits TAA-specific CD8^+^ T cell responses [71]. This strategy has been refined to specifically target DC subsets, such as using DEC205, langerin, and CLEC9A to target cDC1s and CLEC7A to target cDC2s, which is beneficial for specific T cell responses [70]. In a phase I trial in advanced cancers (NCT00948961), CDX-1401—a human anti-DEC-205 antibody fused to the full-length NY-ESO-1 antigen—elicited NY-ESO-1-specific T cell responses in most patients, with some showing clinical benefit, although objective tumor regression was rare [150]. Larger trials are needed to evaluate its potential in early-stage disease and in combination with ICB [150]. Biomaterial scaffolds and nanoparticles facilitate in vivo antigen delivery and are often combined with adjuvants to increase uptake by endogenous DCs [128]. Implantable/injectable biomaterial-based scaffolds enable the controlled release of antigens, chemoattractants, and adjuvants to recruit and activate endogenous DC populations, activating a broader range of DC subsets and continuously providing antigens and stimulus factors [138]. In a phase I trial (NCT01753089), the personalized scaffold vaccine WDVAX—comprising GM-CSF, CpG-ODN, and autologous tumor lysates embedded in a macroporous poly(lactide-co-glycolide) (PLG) matrix scaffold—successfully recruited and activated DCs in situ, inducing immune activation in advanced melanoma patients [151]. Yet, heterogeneity in CD8⁺ T cell and myeloid infiltration, uncertainty regarding antigen interactions within lysates, and the challenge of assessing efficacy in surgical patients awaiting vaccine preparation underscore the need for further optimization [152]. While traditional scaffolds require surgical implantation and repeated dosing, injectable hydrogels are biocompatible and biodegradable with increased delivery efficiency, providing a non-surgical alternative [153]. Nanoparticles reshape the TME, promote DC maturation and T cell infiltration, and can be combined with immune checkpoint inhibitors as an emerging therapeutic strategy [154]. Galactosylated nanoparticles carrying SIINFEKL peptide and CpG-ODNs adjuvant effectively targeted endogenous DCs in mouse models, enhancing DC maturation and T cell recruitment, and underscoring the promise of nanoparticle-based DC vaccines [155]. Additionally, the use of bacteria and viruses as carriers to target DCs with tumor antigens is being explored (e.g., YS-ON-001), these carriers can insert genes encoding TAAs and remove genes encoding autonomous and replicative factors, although pre-existing immunity against vectors may impact vaccine efficacy [148, 156]. Oncolytic herpesvirus talimogene laherparepvec, the BCG vaccine, engineered mitochondria and monophosphoryl lipid A can also serve as tools for DC-targeted antigen delivery [3, 157, 158].

#### Agonists for targeting endogenous DCs

TLRs are PRRs that recognize pathogen-associated molecular patterns (PAMPs) and DAMPs [70]. In humans, cDC1s predominantly express TLR3 and TLR8, cDC2s predominantly express TLR1 and TLR6, and pDCs preferentially express TLR7 and TLR9 [43]. TLR agonists can specifically target DCs, induce their maturation, and enhance their ability to present tumor antigens to T cells [43, 70]. The majority of studies indicate that TLR agonists are safe, well tolerated by patients, and induce immune responses [130]. The cGAS/STING signaling pathway is an innate immune-sensing mechanism activated in response to infection, senescence, DNA damage, and cell cycle dysregulation [70]. STING agonists promote DC maturation, improve antigen presentation, and synergize with anti-PD-1 therapy to strengthen tumor-infiltrating CD8^+^ T cell expansion, showing efficacy in several preclinical models [15, 70, 159]. Optimizing the low bioavailability and delivery methods of traditional STING agonists into tumors and advancing the development and clinical studies of novel non-CDN small-molecule STING agonists are current research priorities [70, 160]. FLT3L is a growth factor that facilitates the proliferation and differentiation of DC precursors in the bone marrow [161]. FLT3L-based therapies (e.g., recombinant soluble FLT3L protein) can enhance antigen presentation and promote immune activation, demonstrating effective synergistic potential with radiotherapy [59]. In a phase I/II trial (NCT01976585), an in situ vaccine combining radiotherapy, intratumoral FLT3L, and the TLR3 agonist Poly(I:C) activated cDC1s, eliciting antitumor CD8⁺ T cell responses and systemic (abscopal) regressions in advanced non-Hodgkin lymphoma [162]. Given the small sample size, response heterogeneity, and absent CD8⁺ T cell immunity in some patients, further studies—particularly in combination with ICB—are warranted to improve efficacy [162]. The activation of CD40 signaling in DCs can upregulate the expression of costimulatory receptors and MHC molecules, enhance antigen presentation, and promote the production of proinflammatory cytokines (e.g., IL-12) and T cell activation [130]. Several CD40 agonists have been developed, but monotherapy with CD40 agonists has shown limited efficacy in treating low-immunogenicity tumors. Multiple clinical evaluations of combination therapies with various CD40 agonists for different cancer types have been conducted [3]. Recent studies have demonstrated that CD40 bispecific antibodies (e.g., CD40-CD11c, CD40-DEC-205, CD40-CLEC9A) can restrict CD40 activation to DCs, thereby retaining antitumor activity while markedly reducing toxicity, expanding the clinical potential of CD40-targeted therapies [163]. In a phase I/II trial (NCT04083599), the bispecific antibody DuoBody-CD40-4-1BB simultaneously engaged CD40 and 4-1BB to activate DCs and T cells, enhancing antitumor immunity [164]. Tumor regression or disease stabilization was observed in some patients, suggesting therapeutic promise, although long-term efficacy, survival benefit, and resistance mechanisms remain to be clarified [164].

### Reversing the immunosuppressive microenvironment to increase the efficacy of DC-Targeted cancer therapy

A primary obstacle in DC-based cancer therapy involves immune suppressive mechanisms established within the TME [165]. Immunosuppressive factors such as TGF-β, IDO, IL-10, VEGF, and PGE2 in the TME impair the maturation and functionality of DCs, enabling immune evasion and tumor progression, and neutralizing these factors promotes DC recruitment, survival, activation, and antigen-presenting capacity [70]. Tumor-derived TGF-β inhibits DC migration to dLNs by reducing CCR7 expression, and TGF-β neutralization via conventional or bispecific antibodies increases functional DC populations in the TME [59, 70]. IDO inhibitors have demonstrated positive effects in murine pancreatic cancer models, with ongoing clinical trials evaluating their combination with DC vaccines in breast cancer (NCT01042535) [34]. Tumor-derived IL-10 impairs DC vaccine efficacy, and blockade of IL-10 production or anti-IL-10R antibody administration synergizes with DC vaccines to suppress tumor growth in tumor-bearing models [32, 166]. VEGF inhibits DC maturation and antitumor immunity, and in several clinical studies, VEGF inhibitors promote DC maturation, enhance immune function, and reduce tumor growth rates [43, 167]. In tumors with high levels of PGE2 expression, combining COX inhibitors with strategies to increase cDC1 numbers has therapeutic benefits [5].

In addition to immunosuppressive factors, immunosuppressive cells such as Tregs and MDSCs infiltrate the TME, impairing DC quantity and function by disrupting DC immune functions and metabolism, thereby promoting cancer progression [52]. The tyrosine kinase inhibitor sunitinib can reduce the abundance of Tregs and MDSCs. In metastatic renal cell carcinoma, a phase II trial (NCT00678119) demonstrated that sunitinib enhanced the immunogenicity of DC vaccines by reducing MDSCs and Tregs, providing a rationale for combinatorial approaches [168]. However, the subsequent phase III ADAPT trial (NCT01582672) showed that although durable immune responses were induced, the combination failed to significantly improve overall survival, underscoring the challenges of clinical translation [169].A phase II trial (NCT02403778) in melanoma patients combined ipilimumab with all-trans retinoic acid to block retinoic acid signaling, forcing MDSC differentiation into macrophages and DCs and suggesting a novel therapeutic approach [170, 171]. TAM survival in the TME depends on CSF-1R signaling [15]. CSF-1R inhibitors disrupt CSF-1/CSF-1R pathways, suppress immunosuppressive TAMs, enhance DC interactions with T/NK cells, and elicit potent T cell-mediated antitumor responses [95].

## Conclusions

DCs serve as pivotal coordinators of antitumor immunity and are capable of recognizing tumor antigens and processing them for presentation to adaptive T cells. DCs exhibit significant heterogeneity within the TME, and the increasing application of new technologies may broaden our understanding of the heterogeneity of DCs in different tumors. Identifying specific biomarkers can help guide subset-selective interventions and identify drugs with targeted immune-stimulatory potential. Current cancer immunotherapies centered around DCs focus mainly on expanding their numbers, activating them, delivering antigens, and enhancing their ability to activate T cells. Endogenous DC-targeted therapies and DC vaccines are most likely integral components of combinatorial regimens, necessitating the determination of the most synergistic therapeutic approaches and their optimal sequencing [3]. As our understanding of the cross-talk between DCs and other cells in the TME deepens, it has been recognized that the immunosuppressive properties of the TME are a major reason why DCs cannot fully exert their immune potential. In the future, the immunosuppressive microenvironment can be reversed through methods such as ICBs, mAbs, ACT therapies, and combination therapies. Moreover, the activity of innate immune cells, such as NK cells, can be enhanced through cytokine supplementation or CAR-NK cells to fully exploit the immune potential of DCs. In addition, the metabolism and changes in DCs in the TME are important for their phenotypic and functional alterations, and targeted therapies against DC metabolic pathways could be used as valuable cancer therapeutic targets in combination with other immunotherapies for cancer treatment. We hope that our work will provide new insights into the application of targeted DCs in future cancer therapy, and we believe that DC-based cancer therapies will show great promise in future cancer treatments.
